# Retraction Note: Bronchial blocker versus double-lumen endobronchial tube in minimally invasive cardiac surgery

**DOI:** 10.1186/s12890-020-01365-7

**Published:** 2020-12-10

**Authors:** Chuncheng Zhang, Jing Yue, Mingyue Li, Wei Jiang, Yu Pan, Zhimin Song, Cailian Shi, Weixuan Fan, Zhenxiang Pan

**Affiliations:** 1 Department of Anesthesiology, The Second Hospital of Jilin University, No.218 Ziqiang Street, Nanguan District, Changchun, 130041 Jilin Province China; 2 Yanbian University, Yanbian, 130000 Jilin province China; 3 Department of Intensive Care Unit, The Second Hospital of Jilin University, Changchun, 130041 Jilin province China

## Retraction Note: BMC Pulm Med 19, 207 (2019) 10.1186/s12890-019-0956-x

The Editor has retracted this article [1] because the authors have been unable to provide documents confirming that ethics approval was obtained in advance of the study. Additionally, discrepancies in study design and recruitment dates were noted between the trial registry [2] and the article.

Authors Chuncheng Zhang, Jing Yue, Mingyue Li, Wei Jiang, Yu Pan, Zhimin Song, Cailian Shi, Weixuan Fan and Zhenxiang Pan all agree to this retraction.
